# Neurotoxic Effects of *trans*-Glutaconic Acid in Rats

**DOI:** 10.1155/2013/607610

**Published:** 2013-03-27

**Authors:** Patrícia F. Schuck, Estela N. B. Busanello, Anelise M. Tonin, Carolina M. Viegas, Gustavo C. Ferreira

**Affiliations:** ^1^Laboratório de Erros Inatos do Metabolismo, Programa de Pós-graduação em Ciências da Saúde, Unidade Acadêmica de Ciências da Saúde, Universidade do Extremo Sul Catarinense, 88806-000 Criciúma, SC, Brazil; ^2^Núcleo de Excelência em Neurociências Aplicadas de Santa Catarina (NENASC), Programa de Pós-Graduação em Ciências da Saúde, Unidade Acadêmica de Ciências da Saúde, Universidade do Extremo Sul Catarinense, 88806-000 Criciúma, SC, Brazil; ^3^Departamento de Bioquímica, Instituto de Ciências Básicas da Saúde, Universidade Federal do Rio Grande do Sul, 90035-003 Porto Alegre, RS, Brazil

## Abstract

*trans*-Glutaconic acid (*t*GA) is an unsaturated C5-dicarboxylic acid which may be found accumulated in glutaric aciduria type I, whose pathophysiology is still uncertain. In the present work it was investigated the *in vitro* effect of increasing *t*GA concentrations on neurochemical and oxidative stress parameters in rat cerebral cortex. We observed that Na^+^, K^+^-ATPase activity was reduced by *t*GA, but not creatine kinase, respiratory chain complex IV, and ATP synthase activities. On the other hand, *t*GA significantly increased lipid peroxidation (thiobarbituric acid-reactive species levels and spontaneous chemiluminescence), as well as protein oxidative damage (oxidation of sulfhydryl groups). *t*GA also significantly decreased nonenzymatic antioxidant defenses (TRAP and reduced glutathione levels). Our data suggest that *t*GA may be neurotoxic in rat brain.

## 1. Introduction

Glutaryl-CoA dehydrogenase deficiency (OMIM: number 231670), also known as glutaric aciduria type I, is an autosomal recessive metabolic disorder due to a blockade in the catabolic pathway of the amino acids lysine, hydroxylysine, and tryptophan. This disease was first described by Goodman et al. [1] and is biochemically characterized by tissue accumulation of predominantly glutaric acid and, to a lesser degree, of 3-hydroxyglutaric and *trans-*glutaconic (*t*GA) acids [2, 3]. Clinically, affected patients present macrocephaly, progressive dystonia, and dyskinesia, symptoms that are apparent within the first year of life [4, 5]. Degeneration of caudate and putamen following encephalopathic crises and frontotemporal atrophy at birth is also commonly seen in these patients [6, 7]. Children presenting residual glutaryl-CoA dehydrogenase activity of up to 30% (low excretors) present low or undetectable excretion of glutaric acid, but the neurological signs may be found similar in these individuals, regardless of the amount of glutaric acid excreted [8–11].

The primary cause of neurological alterations in glutaric acidemia type I is still not defined. It has been demonstrated that brain tissue exposure to the main accumulating metabolites, glutaric, and 3-hydroxyglutaric acids results in excitotoxicity [12–15], oxidative stress induction [16–18], and/or disruption of energy homeostasis [12, 19–24]. Furthermore, it has been recently showed that lysine administration to a knockout mice model of glutaric aciduria type I (*Gcdh−/−*) results in oxidative damage and energy impairment in the brain of these animals, as well as in compromised neurodevelopmental and cognitive behavior [25–28]. On the other hand, very little is known regarding the direct toxicity of *t*GA. In this scenario, and it has been demonstrated that *t*GA is able to inhibits glutamate decarboxylase activity in rat and rabbit brains, human mitochondrial NAD(P)(+)-dependent malic enzyme, and dopamine beta-monooxygenase activities in purified preparations, as well as inducing mitochondrial permeability transition in rat liver mitochondrial preparations, provoking apoptosis in immature oligodendrocytes, and reducing cell viability of primary cultures of mice cerebral neocortical neurons [29–34].

Therefore, the aim of the present work was to investigate the *in vitro* effects of *t*GA on various neurochemical parameters in cerebral cortex of young rats. The investigated parameters were the values of Na^+^, K^+^-ATPase, creatine kinase (CK), complex IV and ATP synthase activities, carbonyl and sulfhydryl content, thiobarbituric acid-reactive substances (TBA-RS) levels, spontaneous chemiluminescence, reduced glutathione (GSH) concentrations, and total nonenzymatic antioxidant capacity of the tissue (TRAP) in cerebral cortex of young rats.

## 2. Material and Methods

### 2.1. Reagents

 All chemicals were purchased from Sigma (St. Louis, MO, USA). *t*GA was dissolved in the day of the experiments in the incubation medium used for each technique, and the solution had its pH adjusted to 7.4. The final concentrations of *t*GA in the medium ranged from 0.01 to 1.0 mM. 

 Parallel experiments were always carried out with negative controls (blanks) in the presence or absence of *t*GA and also with or without cortical supernatants in order to detect artifacts caused by this organic acid in the assays. Therefore, any interference of *t*GA on the reactions used to measure the biochemical parameters would be identified.

### 2.2. Animals

 Thirty-day-old male Wistar rats obtained from the Central Animal House of the Departamento de Bioquímica, Federal University of Rio Grande do Sul (UFRGS), were used. Rats were kept with dams until weaning at 21 days of age. The animals had free access to water and to a standard commercial chow and were maintained on a 12 : 12 h light/dark cycle in an air-conditioned constant temperature (22 ± 1°C) colony room. The “Principles of Laboratory Animal Care” (NIH publication number 80-23, revised 1996) were followed in all experiments and the Ethics Committee for Animal Research of UFRGS, Porto Alegre, Brazil, approved the experimental protocol. All efforts were made to minimize the number of animals used and their suffering. 

### 2.3. Tissue Preparation and Incubation for Oxidative Stress Parameters

 On the day of the experiments the animals were killed by decapitation without anesthesia, and the brain was rapidly excised on a Petri dish placed on ice. The olfactory bulbs, brain stem, medulla, cerebellum, hippocampus, corpus callosum, and striatum were discarded, and the cerebral cortex was peeled away from the subcortical structures, weighed and homogenized in 10 volumes (1 : 10, w/v) of 20 mM sodium phosphate buffer, pH 7.4 containing 140 mM KCl. Homogenates were centrifuged at 750 ×g for 10 min at 4°C to discard nuclei and cell debris [35]. The pellet was discarded, and the supernatant, a suspension of mixed and preserved organelles, including mitochondria, was separated and incubated in 20 mM sodium phosphate buffer, pH 7.4 containing 140 mM KCl at 37°C for one hour with *t*GA at concentrations of 0.01, 0.1, or 1 mM. Controls did not contain this metabolite in the incubation medium. Immediately after incubation, aliquots were taken to measure the values of carbonyl and sulfhydryl content, TBA-RS levels, spontaneous chemiluminescence, GSH concentrations, and TRAP. 

### 2.4. Preparation of Synaptic Plasma Membrane from Rat Cerebral Cortex

 Cerebral cortex was homogenized in 10 volumes of 0.32 mM sucrose solution containing 5.0 mM HEPES and 1.0 mM EDTA. The homogenate was preincubated at 37°C for 1 h in the absence or presence of 0.01, 0.1, or 1 mM *t*GA. Membranes were prepared afterwards according to the method of Jones and Matus [36] using a discontinuous sucrose density gradient consisting of successive layers of 0.3, 0.8, and 1.0 mM. After centrifugation at 69,000 *×*g for 2 h, the fraction at the 0.8–1.0 mM sucrose interface was taken as the membrane enzyme preparation.

### 2.5. Tissue Preparations for Determination of Respiratory Chain Complex IV and Creatine Kinase Activities

 For the determination of the activities of the respiratory chain complex IV, cerebral cortex was homogenized (1 : 20, w/v) in SETH buffer (250 mM sucrose, 2.0 mM EDTA, 10 mM Trizma base, and 50 UI·mL^−1^ heparin), pH 7.4. The homogenates were centrifuged at 800 *×*g for 10 min, and the supernatants were kept at −70°C until being used for enzyme activity determination. For total creatine kinase activity determination, the cerebral cortex was homogenized (1 : 10 w/v) in isosmotic saline solution [37]. 

### 2.6. Mitochondrial Preparations

 Mitochondrial fractions prepared according to Cassina and Radi [38] were used for the determination of ATP synthase activity, TBA-RS levels, and sulfhydryl content.

### 2.7. Determination of Na^+^, K^+^-ATPase Activity

 Na^+^, K^+^-ATPase activity was evaluated according to Tsakiris and Deliconstantinos [39]. Released inorganic phosphate (Pi) was measured by the method of Chan et al. [40]. Enzyme-specific activity was expressed as nmol Pi released·min^−1^·mg protein^−1^.

### 2.8. Determination of Bioenergetics Parameters

 The activity of respiratory chain complex IV was determined according to Rustin et al. [41] slightly modified, as described in details in a previous report [42]. ATP synthase activity was measured in mitochondrial preparations from cerebral cortex, according to Rustin et al. [41]. Complex IV and ATP synthase activities were calculated as nmol·min^−1^·mg protein^−1^.

 CK activity was measured in total homogenates according to Hughes [43] with slight modifications, as described by Schuck et al. [37]. Results are expressed as *μ*mol of creatine·min^−1^·mg protein^−1^.

### 2.9. Determination of Protein Carbonyl Formation Content

 Protein carbonyl content, a marker of oxidized proteins, was measured spectrophotometrically according to Reznick and Packer [44]. The results were calculated as nmol of carbonyls groups·mg of protein^−1^, using the extinction coefficient of 22,000 × 106 nmol·mL^−1^ for aliphatic hydrazones.

### 2.10. Sulfhydryl (Thiol) Group Oxidation

 This assay was performed according to Aksenov and Markesbery [45]. The protein-bound sulfhydryl content is inversely correlated to oxidative damage to proteins. Results were reported as nmol TNB·mg of protein^−1^.

### 2.11. Determination of TBA-RS Levels

 TBA-RS was determined according to the method of Esterbauer and Cheeseman [46]. A calibration curve was performed using 1,1,3,3-tetramethoxypropane, and each curve point was subjected to the same treatment as supernatants. Some experiments were performed in the absence or presence of reduced glutathione (GSH; 100 *μ*M), melatonin (100 *μ*M), the nitric oxide synthase inhibitor N^*ω*^-nitro-L-arginine methyl ester (L-NAME; 500 *μ*M), trolox (5 *μ*M), or a combination of catalase (Cat; 10 mU·mL^−1^) plus superoxide dismutase (SOD; 10 mU·mL^−1^). TBA-RS values were calculated as nmol of TBA-RS·mg protein^−1^.

### 2.12. Spontaneous Chemiluminescence

 Samples were assayed for spontaneous chemiluminescence in a dark room by the method of Gonzalez-Flecha et al. [47]. Results were calculated as cpm·mg protein^−1^.

### 2.13. Determination of TRAP

 TRAP, representing the total nonenzymatic antioxidant capacity of the tissue, was determined by measuring the chemiluminescence intensity of luminol induced by 2,2′-azo-bis-(2-amidinopropane) (ABAP) according to the method of Lissi et al. [48]. TRAP values were expressed as nmol of trolox·mg of protein^−1^.

### 2.14. GSH Levels Quantification

 GSH levels were measured according to Browne and Armstrong [49]. Some experiments were performed in the presence or absence of melatonin (100 *μ*M), L-NAME (500 *μ*M), trolox (5 *μ*M), or Cat (10 mU·mL^−1^) plus SOD (10 mU·mL^−1^). Calibration curve was prepared with standard GSH (0.01–1 mM), and the concentrations were calculated as nmol·mg protein^−1^.

 The oxidation of a commercial solution of GSH (200 *μ*M) was also tested by exposing this solution to 1 mM *t*GA for one hour in a medium devoid of brain supernatants. After *t*GA exposition, 7.4 mM *o*-phthaldialdehyde was added to the vials, and the mixture was incubated at room temperature during 15 minutes.

### 2.15. Protein Determination

 Protein was measured by the method of Lowry et al. [50] using bovine serum albumin as standard.

### 2.16. Statistical Analysis

 Results are presented as mean ± standard deviation. Assays were performed in duplicate, and the mean was used for statistical analysis. Data was analysed using one-way analysis of variance (ANOVA) followed by the post hoc Duncan multiple range test when *F* was significant. Differences between groups were rated significant at *P* < 0.05. All analyses were carried out in an IBM-compatible PC computer using the Statistical Package for the Social Sciences (SPSS) software.

## 3. Results

*t*GA inhibits Na^+^, K^+^-ATPase activity in synaptic plasma membranes from rat cerebral cortex.

**Figure 1:**
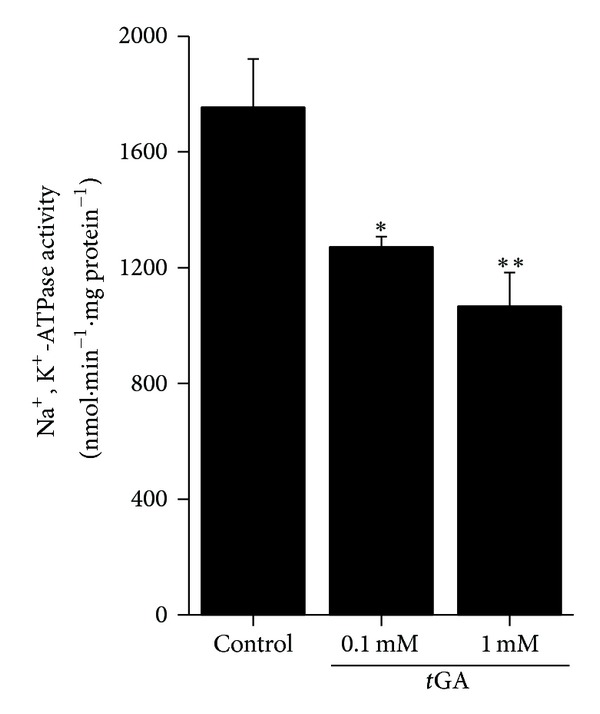
*In vitro* effect of *trans-*glutaconic acid (*t*GA) on Na^+^, K^+^-ATPase activity in rat cerebral cortex of 30-day-old rats. The experiments were performed in triplicate, are plotted as mean ± S.E.M. (*n* = 4), and are expressed as nmol·mg protein^−1^. *P < 0.05 and **P < 0.01 compared to control group (Duncan multiple range test).

Initially, we tested the influence of *t*GA on Na^+^, K^+^-ATPase activity in synaptic plasma membranes prepared from cerebral cortex.  Figure 1 shows that the incubation of *t*GA with purified synaptic membrane preparations caused a significant inhibition of Na^+^, K^+^-ATPase activity. 

### 3.1. *t*GA Does Not Interfere on Important Parameters of Brain Bioenergetics

 We also investigated the effect of *t*GA on important parameters of brain bioenergetics. It is observed in Table 1 that the presence of up to 1.0 mM *t*GA in the incubation medium does not disturb creatine kinase, respiratory chain complex IV, and ATP synthase activities in rat cerebral cortex.

### 3.2. Protein Oxidative Damage Is Induced by *t*GA

**Figure 2:**
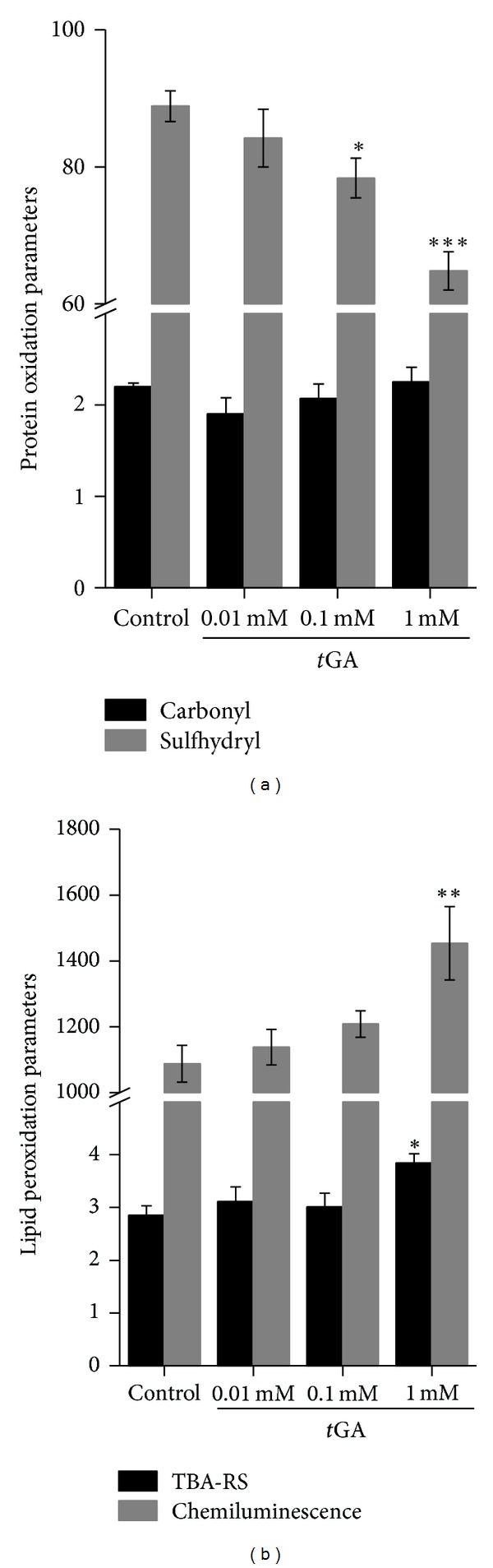
*In vitro* effect of *trans-*glutaconic acid (*t*GA) on oxidative damage parameters (a) carbonyl and sulfhydryl content and (b) TBA-RS levels and spontaneous chemiluminescence in cerebral cortex of 30-day-old rats. The experiments were performed in triplicate and are plotted as mean ± S.E.M. (*n* = 5-6). Carbonyl and sulfhydryl content and TBA levels are expressed as nmol·mg protein^−1^; chemiluminescence values are expressed as cpm·mg protein^−1^. *P < 0.05; **P < 0.01 and ***P < 0.001 compared to control group (Duncan multiple range test).

We then evaluated the effect of *t*GA on carbonyl formation and sulfhydryl oxidation in cortical homogenates in order to evaluate protein oxidative damage. We found that *t*GA provoked a significant decrease of sulfhydryl content at 0.1 and 1.0 mM in cortical homogenates (Figure 2(a)), although it did not affect protein carbonyl content. Furthermore, sulfhydryl content was not altered by *t*GA in brain mitochondrial preparations (Table 2). 

### 3.3. *t*GA Induces Lipid Peroxidation

The influence of *t*GA on the lipid peroxidation parameters TBA-RS levels and spontaneous chemiluminescence was investigated.  Figure 2(b) shows that *t*GA at 1 mM significantly increased TBA-RS levels in cortical homogenates. Similar results were obtained for spontaneous chemiluminescence.

**Figure 3:**
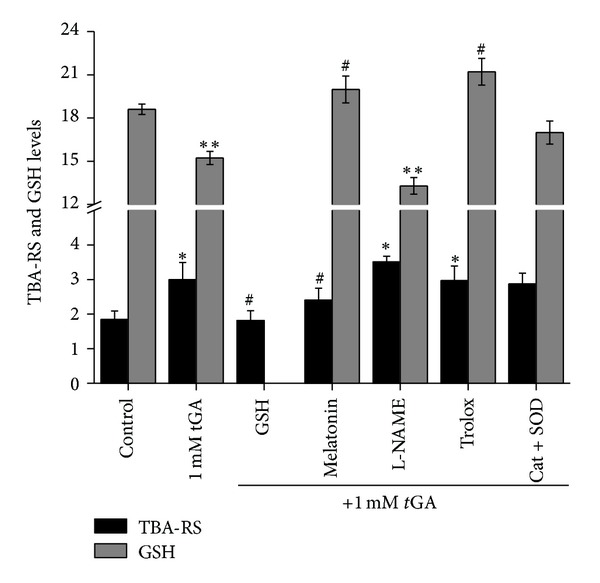
*In vitro *effect of the antioxidants GSH (100 *μ*M), melatonin (100 *μ*M), L-NAME (500 *μ*M), trolox (5 *μ*M), and catalase (Cat) plus superoxide dismutase (SOD; 10 mU·mL^−1^ each) on *trans*-glutaconic- (*t*GA-) induced alterations on thiobarbituric-acid reactive substances (TBA-RS) and glutathione (GSH) levels in cerebral cortex of 30-day-old rats. Cortical homogenates were preincubated for 15 min with the antioxidants before the addition of the 1 mM *t*GA. The experiments were performed in triplicate, are plotted as mean ± S.E.M. (*n* = 4-5), and are expressed as nmol·mg protein^−1^. *P < 0.05 and **P < 0.01 compared to control group; ^#^ *P* < 0.05 compared to *t*GA group (Duncan multiple range test).

We also tested the effect of the antioxidants GSH (100 *μ*M), melatonin (100 *μ*M), L-NAME (200 *μ*M), trolox (5 *μ*M), or a combination of Cat plus SOD (10 mU mL^−1^ each) on *t*GA-elicited increase of TBA-RS levels (Figure 3). These antioxidants were coincubated with 1.0 mM of the organic acid and brain cortical homogenates for 60 min, after which the levels of TBA-RS were measured. We observed that GSH and melatonin fully prevented *t*GA-induced increased lipid peroxidation, whereas the combination of Cat plus SOD partially prevented such effects. Trolox and L-NAME did not prevent *t*GA effect.

It was also observed that *t*GA did not affect TBA-RS levels in brain mitochondrial preparations (Table 2).

### 3.4. Nonenzymatic Antioxidant Defenses Are Decreased by *t*GA

**Figure 4:**
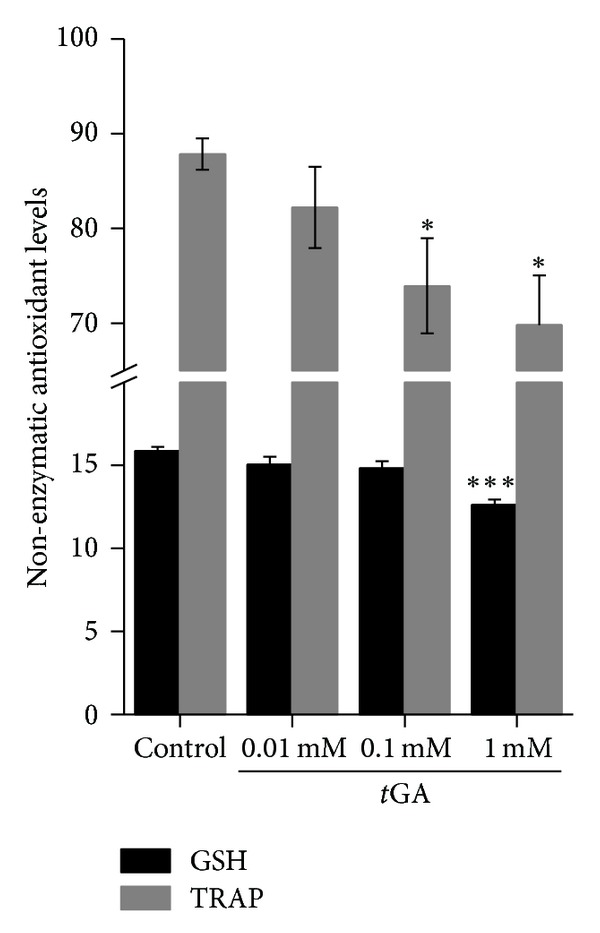
*In vitro* effect of *trans-*glutaconic acid (*t*GA) on the nonenzymatic antioxidant markers *total-*radical trapping antioxidant potential (TRAP) and glutathione (GSH) levels in rat cerebral cortex of 30-day-old rats. The experiments were performed in triplicate, plotted as mean ± S.E.M. (*n* = 5-6), and are expressed as nmol·mg protein^−1^. *P < 0.05 and ***P < 0.001 compared to control group (Duncan multiple range test).

We assessed the *in vitro* effect of *t*GA on cortical GSH levels and TRAP. We found that this organic acid significantly decreased GSH levels and TRAP at the highest tested concentration (1.0 mM) (Figure 4). Furthermore, *t*GA-induced effect was totally prevented by the free-radical scavengers melatonin and trolox and by the combination of Cat plus SOD, but not by L-NAME (Figure 3).

We then investigated whether *t*GA-induced GSH levels decrease was secondary to a direct oxidative attack. We therefore exposed a commercial solution of GSH (200 *μ*M) to 1.0 mM *t*GA for 60 min in the absence of cortical supernatants. It was observed that this organic acid *per se* did not oxidize highly purified GSH (Table 2).

## 4. Discussion

*t*GA, or (*E*)-pentene-1,5-dioic acid, is an unsaturated C5-dicarboxylic acid that is found in patients affected by glutaric aciduria type I. Its role on this organic aciduria pathogenesis is under debate, since there are patients affected by glutaric aciduria type I who do not present *t*GA accumulation [3]. On the other hand, several *t*GA toxic effects have already been reported [29–34].

Herein we present data demonstrating that Na^+^, K^+^-ATPase activity, a crucial enzyme for maintaining ion homeostasis and membrane potential in cells [51], was inhibited by *t*GA at 0.1 and 1.0 mM concentrations. Inhibition of Na^+^, K^+^-ATPase activity has been described in various diseases, including cerebral ischemia [52, 53], neurodegenerative [54], and metabolic disorders [55, 56]. Furthermore, studies have indicated that Na^+^, K^+^-ATPase inhibition may result in cellular death by activating apoptotic cascades and neuronal damage probably due to amplification of potassium homeostasis impairment [57]. 

Considering that this enzyme is present at high concentrations in brain cellular membranes and consumes about 40–50% of the ATP generated [58], our next step was to evaluate the effects of *t*GA on some important bioenergetics parameters in order to identify whether ATP deficit could contribute to Na^+^, K^+^-ATPase activity inhibition caused by this organic acid. It was observed that *t*GA does not affect the activities of respiratory chain complex IV, creatine kinase, and ATP synthase. However, at this point, it should be mentioned that effects of *t*GA on glycolysis Krebs cycle enzyme activities, and the other respiratory chain complexes activities cannot be ruled out. 

It was further investigated the *in vitro* effect of *t*GA on oxidative stress parameters, since Na^+^, K^+^-ATPase activity is highly dependent of critical sulfhydryl groups present in its catalytic site, rendering this enzyme activity highly susceptible to oxidative damage [59, 60]. We showed that *t*GA *in vitro* induced oxidative damage to proteins, as observed by sulfhydryl content decrease, and to lipids, since it increased TBA-RS levels and spontaneous chemiluminescence. Interestingly, carbonyl content was not altered by the presence of *t*GA in the incubation medium, suggesting that particularly sulfhydryl groups are prone to protein oxidative damage by this organic acid. On the other hand, light emitted in the spontaneous chemiluminescence assay mainly arises from oxidized lipids due to an increase in reactive oxygen or nitrogen species production, and TBA-RS levels reflects the amount of malondialdehyde formation, an end product of membrane fatty acid peroxidation [61]. In this scenario, it is tempting to speculate that *t*GA oxidized lipids from cell and organelle membranes, which could lead to an impairment of membrane fluidity. Consequently, integral proteins such as Na^+^, K^+^-ATPase could also be affected, having their functioning impaired.

Furthermore, it was observed that GSH and melatonin fully prevented *t*GA-induced increased lipid peroxidation, since these compounds prevented TBA-RS increase elicited by this organic acid, whereas the combination of Cat plus SOD only partially prevented such effects. On the other hand, trolox (a hydrophilic vitamin E analogue) and L-NAME did not prevent *t*GA effect. It is speculated that lipid oxidation induced by *t*GA occurs due to an increase of reactive oxygen species.

We then assessed nonenzymatic antioxidant defenses by measuring GSH levels and TRAP and observed that both parameters were decreased by *t*GA *in vitro*. Since these parameters are suitable to evaluate the capacity of a tissue to prevent and respond to oxidative damage [35, 61, 62], it is likely that *t*GA impairs rat cortical antioxidant defenses. Moreover, *t*GA-induced effect on GSH levels was totally prevented by the free-radical scavengers melatonin and trolox and by the combination of Cat plus SOD, but not by L-NAME, suggesting that mainly peroxyl, alkoxyl, and hydroxyl radicals were involved in the reduction of GSH levels provoked by *t*GA. Considering that *t*GA was not able to oxidize a commercial GSH solution in the absence of brain supernatants in the incubation medium, it is unlikely that this organic acid is *per se *a direct oxidant agent, corroborating the idea that it probably provoked lipid and protein oxidative damage by increasing free radical generation.

Since the alterations elicited by *t*GA on TBA-RS and sulfhydryl content in whole homogenates, which contain the whole cell machinery, were not observed when this organic acid was supplemented to mitochondrial preparations, it is feasible that oxidative stress induced by *t*GA was probably mediated by cytosolic mechanisms. For instance, some putative mechanisms could involve xanthine oxidase, cytosolic NADPH oxidase, lysosomes, peroxisomes, and others [63].

Oxidative stress is an imbalance between the total tissue antioxidant (enzymatic and nonenzymatic) defenses and reactive species generation in cell leading to a deleterious cell condition [61]. In this study, we showed evidences that *t*GA induces oxidative stress in cerebral cortex *in vitro*, since it increased oxidative damage and decreased antioxidant defenses. It should be emphasized that the brain is highly susceptible to damage induced by increased reactive species generation, since it has low cerebral antioxidant defenses as compared to other tissues [64]. Furthermore, it may be also speculated that the inhibition of Na^+^, K^+^-ATPase activity elicited by *t*GA reported in the present study could be secondary to increased oxidation of critical sulfhydryl groups at the catalytic site of the enzyme.

Taken together, our results provide evidences that *t*GA is toxic to brain cells *in vitro*, by causing alterations in cell ion balance, and probably neurotransmission, as well as oxidative stress in rat cerebral cortex. We cannot at this point establish whether our data have a pathophysiological significance for glutaric aciduria type I. *t*GA accumulates in urine for excretion [65, 66], and excretion of this acid may become prominent, exceeding that of 3-hydroxyglutaric acid, during episodes of ketosis [4, 67]. Some studies indicated that the diagnostic relevance of *t*GA is limited [68], since *t*GA excretion in urine may be inconsistently found. However, it should be highlighted that even glutaric acid, known to be the major accumulating in glutaric aciduria type I, may be excreted at discrete or even normal concentrations (low excretor patients), as above mentioned. 

## 5. Conclusion

Considering that the main signs and symptoms of affected patients are neurological [3], and in case the present findings are confirmed *in vivo* in animal experiments and also in tissues of patients accumulating *t*GA, it may be speculated that *t*GA toxicity could collaborate to the brain damage characteristic of glutaric aciduria type I affected patients. 

## Figures and Tables

**Table 1:** *In vitro* effect of *trans*-glutaconic acid (*t*GA) on creatine kinase (CK), respiratory chain complex IV, and ATP synthase activities in cerebral cortex of 30-day-old rats.

Group	CK	Complex IV	ATP synthase
Control	11.28 ± 0.20	114.72 ± 7.42	476.00 ± 31.23
0.1 mM *t*GA	10.66 ± 0.78	116.46 ± 2.57	—
1 mM *t*GA	11.04 ± 1.18	107.14 ± 3.29	508.75 ± 15.47

The experiments were performed in triplicate, and data represent mean ± standard error of the mean (*n* = 4-5). It was not observed any difference among groups (ANOVA and Student' *t*-test). CK: *μ*mol · min^−1^ · mg of protein^−1^; complex IV and ATP synthase: nmol · min^−1^ · mg of protein^−1^.

**Table 2:** *In vitro* effect of *trans*-glutaconic acid (*t*GA) on thiobarbituric acid-reactive species (TBA-RS) levels and sulfhydryl (SH) content in mitochondrial preparations from cerebral cortex of 30-day-old rats and glutathione (GSH) content in a commercial GSH preparation.

Group	TBA-RS	SH	GSH
Control	6.44 ± 0.59	55.98 ± 2.30	2941 ± 144.3
1 mM *t*GA	6.16 ± 0.40	56.22 ± 3.59	2640 ± 70.35

The experiments were performed in triplicate, and data represent mean ± standard error of the mean (*n* = 4-5). It was not observed any difference between groups (Student' *t*-test for paired samples). TBA-RS and SH: nmol · mg of protein^−1^; GSH: fluorescent unities.
